# Protocol for Factors Influencing Burnout and Its Impact on Nurse Retention in the Selected District Hospital, Eastern Cape

**DOI:** 10.3390/ijerph23020214

**Published:** 2026-02-09

**Authors:** Ezile Ndamase, Eric Maimela, Ntiyiso Vinny Khosa

**Affiliations:** 1 School of Public Health, Faculty of Medicine and Health Sciences, Walter Sisulu University, Mthatha 5117, South Africa; 216039770@mywsu.ac.za (E.N.); emaimela@wsu.ac.za (E.M.); 2 WSU Institute for Clinical Governance and Healthcare Administration, School of Public Health, Walter Sisulu University, East London 5200, South Africa; 3 Biostatistics and Analytics Training Services Unit, School of Medicine and Health Sciences, Walter Sisulu University, East London 5200, South Africa; 4 Division of Epidemiology and Biostatistics, School of Medicine and Health Sciences, Walter Sisulu University, East London 5200, South Africa

**Keywords:** burnout, hospital, factors, nurse retention

## Abstract

Burnout among nurses remains a major challenge in South Africa’s public healthcare system, particularly in district hospitals where high workloads, staff shortages, and limited resources are common. In the Eastern Cape, the demands of rural healthcare further exacerbate stress, leading to emotional exhaustion, reduced professional accomplishment, and high turnover rates. This study aims to investigate the factors influencing burnout and its impact on nurse retention at St. Elizabeth Hospital in the Eastern Cape. A quantitative, cross-sectional design will be employed using stratified random sampling, with hospital departments serving as strata. A total of 209 nurses will be selected using Slovin’s formula. Data will be collected through a structured questionnaire incorporating the Burnout Assessment Tool, Maslach Burnout Inventory, Personal Accomplishment Subscale, and a Burnout Knowledge and Response Survey. Descriptive statistics will summarize demographic and burnout characteristics, while chi-square tests, *t*-tests/ANOVA, Pearson correlation, and multiple regression analyses will identify associations and predictors. The study will generate evidence-based insights to guide the development of targeted interventions, including workload redistribution, enhanced managerial support, and wellness initiatives. Ultimately, improving nurse retention will enhance workforce stability, promote staff well-being, and strengthen the quality of patient care in Eastern Cape district hospitals.

## 1. Introduction

Nurse retention is a critical issue in healthcare systems worldwide, particularly in district hospitals where healthcare demands are high and resources are often limited in developing countries. Thus, burnout is one of the main factors influencing nurse retention, which can significantly impact the emotional, physical, and mental well-being of nurses, leading to decreased job satisfaction, high turnover, and compromised patient care [1]. These burnout issues are multifaceted, involving dimensions such as emotional exhaustion, depersonalization, and reduced personal accomplishment, all of which contribute to the declining quality of healthcare services [2]. The relationship between nurse burnout and retention rates requires urgent investigation. This chapter will introduce the problem of burnout in district hospitals, focusing on its effects on nurse retention.

### 1.1. Background

Burnout among healthcare professionals, particularly nurses, is a well-documented issue that negatively affects both the individuals experiencing it and the healthcare system. Recent studies have demonstrated a direct link between burnout and high nurse turnover rates, which have become a pressing concern for hospitals across South Africa [3]. Compounding this problem, government policies that have frozen hiring for new nursing positions and halted the intake of community service nurses have further strained the system, exacerbating turnover challenges [4].

The Eastern Cape province has an estimated population of approximately 6.7 million people [5]. According to the South African Nursing Council [6] and the Eastern Cape Department of Health [7], the nursing workforce consists of 11,871 professional nurses, 5404 nursing auxiliaries, and 3494 staff nurses (enrolled nurses). Professional nurses possess full clinical and administrative training, enrolled nurses have a more limited scope of practice, and nursing auxiliaries provide basic patient care under supervision.

The World Health Organization (WHO) recommends a minimum nurse-to-population ratio of one nurse per 1000 people, encompassing the total nursing and midwifery workforce rather than only professional nurses [8]. For the Eastern Cape’s population, this translates to a minimum of 6700 nurses. Based on ECDoH data, the province currently employs 20,769 nurses across all categories, yielding an overall ratio of approximately one nurse per 323 people, or about three nurses per 1000 people.

Focusing solely on professional nurses reveals a more constrained capacity. With 11,871 professional nurses serving the population, the ratio decreases to roughly one professional nurse per 565 people, or about 1.77 professional nurses per 1000 people. This significant difference between overall and professional nurse ratios points to a critical shortage in the cadre most responsible for clinical care and leadership.

Workforce distribution plays a pivotal role in healthcare access and quality, as highlighted by [6,8]. In the Eastern Cape, there is a tendency for nurses to be concentrated in urban and tertiary care settings, while rural district hospitals and clinics face ongoing staffing shortages [9]. Given that professional nurses bear the heaviest clinical and managerial workloads, shortages in this category particularly affect high-acuity and rural environments, contributing to increased workloads, burnout, and turnover [8].

Furthermore, official workforce statistics do not always reflect the active clinical nursing capacity, since some registered nurses may be on leave, engaged in training, retired, or employed in administrative roles [6]. This discrepancy between registered personnel and operational staff exacerbates staffing shortfalls and places additional pressure on those available.

Research consistently underscores a strong association between job stress and high turnover intentions among nurses [10,11]. The demanding nature of nursing, requiring intense physical and emotional labor, makes burnout particularly prevalent, with reported rates ranging from 10% to 70%, and higher prevalence observed in sub-Saharan Africa [12]. In district hospitals, where nurse-to-patient ratios are frequently skewed, burnout effects are especially severe. [1] found that moderate burnout was common among 54% of nurses studied, and emotional exhaustion was significantly correlated with turnover intentions; specifically, each one-unit increase in emotional exhaustion was linked to a 12% higher likelihood of leaving the job [1]. This dynamic creates a vicious cycle in which burnout fuels turnover, further burdening the remaining staff and perpetuating retention challenges [13].

Nurse retention remains a critical issue within South Africa’s healthcare system, particularly in the Eastern Cape, where district hospitals continue to face substantial staffing shortages due to hiring freezes and suspension of community service nurse intakes [14]. These factors intensify pressure on current nursing staff, aggravating burnout and negatively impacting retention.

According to Eastern Cape Department of Health statistics for 2024, retention rates stand at 88.7% for professional nurses, 95.2% for assistant nurses, and 93.5% for staff nurses. While these figures indicate progress in retaining nurses, vacancy rates remain problematic. Professional nurse vacancies are reported at 11.0%, assistant nurses at 4.8%, and staff nurses at 6.5%, highlighting ongoing staffing gaps. Notably, the professional nurse vacancy rate exceeds the commonly accepted threshold of 10%, which is associated internationally with increased workloads, burnout, and higher turnover [1,15]. Given the persistent shortages and high burnout prevalence, there is an urgent need to explore the factors influencing burnout and their impact on nurse retention in district hospitals within the Eastern Cape [16].

Previous studies conducted in South African district and regional hospitals indicate high levels of nurse burnout, particularly emotional exhaustion, with prevalence estimates ranging from 45% to over 60% [1,12,17]. These findings suggest that burnout is already a significant concern within comparable healthcare settings, underscoring the need to systematically explore its determinants and impact on nurse retention at St. Elizabeth Hospital.

### 1.2. Problem Statement

The problem is compounded by limited research on the unique challenges faced by district hospitals in South Africa, especially in the Eastern Cape, where resource limitations and high patient loads exacerbate the burnout experience among nurses. Therefore, it is necessary for this study to be conducted to investigate factors influencing burnout on nurse retention in the selected district hospital in the Eastern Cape. This study aims to investigate the impact of burnout on nurse retention at selected district hospitals in the Eastern Cape, South Africa. Burnout among nurses characterised by emotional exhaustion, depersonalisation, and reduced personal accomplishment has been widely associated with decreased job satisfaction and increased staff turnover [1]. However, the specific relationship between burnout and retention within the context of district hospitals in the Eastern Cape remains underexplored. This is particularly concerning given recent developments, such as the freezing of nursing posts and the suspension of community service placements, which have led to critical staffing shortages and increased workload for remaining nurses.

These conditions place significant strain on the healthcare workforce, contributing to physical and emotional fatigue that may drive nurses to leave the profession or seek work elsewhere. The situation is further worsened by the lack of research focused on the unique operational and structural challenges faced by district hospitals in South Africa. This absence of locally relevant evidence makes it more difficult to develop effective, targeted interventions and policies, thereby intensifying, or *compounding*, the existing problem. Without a clear understanding of how burnout influences retention in these settings, decision-makers remain ill-equipped to address workforce sustainability.

Therefore, this study is both timely and necessary. It will contribute to filling the current knowledge gap by exploring the specific factors that contribute to burnout and how these influence nurse retention in selected district hospitals within the Eastern Cape.

### 1.3. Justification of the Study and Significance

This study is significant as it aims to provide valuable insights into the effects of burnout on nurse retention at a selected Hospital, a district hospital facing significant staffing challenges. Understanding this relationship is crucial for developing targeted interventions to mitigate burnout and enhance nurse retention. The decision to close nursing posts and discontinue the intake of community service nurses in South Africa is largely driven by financial constraints and national fiscal pressures faced by the government [4]. However, while these measures may offer short-term financial relief, they do not account for the long-term impact on the nursing workforce.

The closure of nursing posts and the reduction in nurse intake may lead to staffing shortages, further contributing to burnout and dissatisfaction within the profession [16]. Current healthcare policies may not fully prioritise nurse welfare and retention, resulting in a cycle where the lack of support for nurses increases turnover rates, putting additional strain on the system [17]. This study is particularly timely as it explores how these factors influence nurse retention in a district hospital in the Eastern Cape province, which are already struggling to maintain an adequate and effective workforce amid rising burnout and staff shortages.

Addressing burnout and retention is crucial for improving the well-being of nurses and the quality of care provided to patients [13]. The findings of this study could inform policymakers to make decisions that will lead to improved working conditions for nurses, better retention strategies, and enhanced healthcare outcomes for patients [14]. Furthermore, by addressing the cycle of burnout, retention, and patient care, this research can contribute to the long-term sustainability of healthcare services at a selected district hospital.

### 1.4. Research Questions

What factors contribute to burnout among nurses, and how does burnout influence nurse retention at the selected district hospital?

### 1.5. Research Objectives

*Overall Objective*

To investigate factors influencing burnout and its impact on nurse retention at a selected district hospital in the Eastern Cape.

*Specific Objectives*

1. To determine the level of burnout among nurses at the selected hospital.
2. To identify organisational and individual factors contributing to nurse burnout.
3. To examine the relationship between burnout and nurse retention.
4. To assess nurses’ knowledge and awareness of burnout and institutional support strategies.
5. To propose evidence-based recommendations to improve nurse retention.

## 2. Materials and Methods

### 2.1. Study Design

This study will use a quantitative research design to examine the relationship between burnout and its impact on nurse retention at St. Elizabeth’s Hospital in Eastern Cape Province. By utilizing a quantitative approach, the study can provide statistical evidence of the impact of burnout on nurse retention, making it easier to generalize the findings to similar district hospitals in South Africa. According to [18] quantitative research provides an objective framework for testing hypotheses and examining the relationships between variables, making it an appropriate choice for this study [15]. Furthermore, quantitative methods allow for efficient data collection within a short space of time, from a large sample of participants, which is crucial in ensuring the generalizability of the findings.

A cross-sectional survey will be conducted to gather data on the burnout levels among nurses, retention rates, and patient outcomes at a single point in time. This allows the researcher to collect data efficiently once. A cross-sectional survey is particularly useful in identifying patterns and relationships between variables at one point in time [18]. In this case, the survey will assess nurse burnout and its impact on retention. This approach is in line with the study objectives. The quantitative cross-sectional design aligns with the study objectives by allowing measurement of burnout levels, identification of contributing factors, assessment of associations with nurse retention, and examination of predictors within a defined population at a single point in time. This study is not intended to establish causality but rather to describe burnout levels and examine associations with nurse retention within a district hospital context.

### 2.2. Study Setting

The study will be conducted at St. Elizabeth Hospital, a district hospital located in the Eastern Cape province of South Africa. The Eastern Cape province was chosen due to its significant healthcare challenges, including high patient volumes and considerable strain on clinical staff and infrastructure. District hospitals in this region are key components of the public healthcare system, providing essential services such as emergency care, maternity care, general medical services, and surgical interventions. St. Elizabeth Hospital was purposively selected because it is a large district hospital serving a predominantly rural population, with persistently high patient volumes, documented nursing shortages, and reliance on professional nurses to deliver both clinical and managerial services. These characteristics make it a representative and information-rich setting for examining burnout and nurse retention challenges typical of district hospitals in the Eastern Cape.

### 2.3. Population

The population for this study, consisting of all employed nurses at St. Elizabeth Hospital on a full-time basis who are professional nurses, staff nurses, and auxiliary nurses for the 2025 financial year, is of significant relevance and applicability to the field of nursing and healthcare management. In this study, professional nurses refer to nurses registered with SANC with comprehensive clinical and managerial responsibilities, while staff nurses (enrolled nurses) provide nursing care within a more limited scope of practice under supervision. This distinction is consistent with South African nursing classifications.

### 2.4. Sampling Method

To ensure that the sample accurately represents the population of nurses at St. Elizabeth Hospital, stratified random sampling will be used. Each department will be treated as a stratum. This method will ensure that nurses from different departments, such as emergency, maternity, general medical, surgical, and paediatrics, are adequately represented [19]. The nurses will further be divided into professional nurses, staff nurses, and auxiliary nurses. Within each stratum, participants will be selected using a computer-generated random number list based on the staff register, ensuring transparency and minimizing selection bias.

### 2.5. Sample Size Calculation

The total sample size of the study will be calculated using Slovin’s formula below. The population size, which makes the sum of 362, was added with a value of 1 and multiplied by the level of error, 0.052. Then, after, the population size, which is the denominator, was divided by the numerator obtained after adding the population size to the value 1 and multiplying by 0.052. Slovin’s formula was selected because it is appropriate for estimating sample size in finite populations when population variance is unknown. Stratification by department and professional category was used to address population heterogeneity.

The sample size was calculated using Slovin’s formula below

$$n=\frac{N}{1+Ne^{2}}$$

*n* = sample size of the adjusted population
N = population size
E = accepted level of error, usually set at 0.05

$$n=\frac{N}{1+Ne^{2}}$$

$$n=\frac{362}{1+362\times 0.0025}$$

$$n=\frac{362}{1.905}$$

n=190

To account for potential non-response and incomplete questionnaires, a 10% inflation (*n* = 19) was added, resulting in a final sample size of 209 nurses. A 10% adjustment is commonly applied in survey-based studies to maintain adequate statistical power.

We will round up to the nearest whole number to ensure sufficient statistical power: *n* = 190. Therefore, 10% (19) will be added to cater to non-respondents and incomplete responses N = 209.

### 2.6. Inclusion and Exclusion Criteria

#### 2.6.1. Inclusion Criteria

Professional nurses, staff nurses, and auxiliary nurses who are employed on a full-time basis at St. Elizabeth Hospital with one year or more of working experience will be included in this study.

#### 2.6.2. Exclusion Criteria

Nurses who are working as part of community service will be excluded because their workload exposure, shift patterns, and contractual obligations differ substantially from those of full-time nurses, which may confound burnout measurement and retention indicators.

### 2.7. Study Procedure

The study will be conducted in several sequential phases, beginning with obtaining ethical approval and culminating in data collection and analysis.

Ethical Approval: Upon receiving ethical clearance from Walter Sisulu University’s Ethics Review Committee, the research team will prepare all necessary documentation, including the study protocol, informed consent forms, and participant information sheets. The approval will be formally documented and used to guide subsequent steps. This study involves minimal researcher intervention, as data will be collected using self-administered questionnaires without manipulation of the work environment or participant behaviour.

### 2.8. Recruitment

Following ethical approval, the research team will liaise with the management of the selected district hospitals in the Eastern Cape to facilitate participant recruitment. Nurses will be informed about the study through hospital communication channels, such as staff meetings, notice boards, and email notifications. Information sessions will be held to explain the purpose of the study, what participation entails, and to address any questions. Participation will be voluntary, and all nurses will be assured of confidentiality and the right to withdraw at any time without penalty. Informed consent will be obtained from each participant prior to data collection.

### 2.9. Data Collection Preparation

Before data collection begins, questionnaires and surveys will be prepared, piloted with a small sample of nurses from a similar setting to ensure clarity and appropriateness. Necessary materials, such as printed questionnaires and consent forms, will be organized, and data collection teams will be trained on standardized procedures to ensure consistency.

### 2.10. Data Collection

Data will be collected over a specified period, for example, 4–6 weeks, to accommodate nurses’ schedules and to avoid interrupting hospital activities. During this period:

Nurses will complete the Maslach Burnout Inventory (MBI), a validated self-administered questionnaire measuring burnout across three dimensions: emotional exhaustion, depersonalization, and reduced personal accomplishment [20] as cited by [21,22].

Additionally, participants will complete the Burnout Knowledge and Response Survey, designed to assess their understanding of burnout, its symptoms, causes, and impacts on retention, as well as their perceptions of institutional support and personal coping strategies [23,24].

The questionnaires will be distributed in paper form. Participants will be given adequate time to complete the surveys in a quiet, private setting to ensure honest and thoughtful responses.

### 2.11. Retention Data

Concurrently, data on nurse retention rates will be obtained from the hospital’s human resources records. This data will include the number of nurses who leave the hospital during the study period, providing quantitative retention indicators. Nurse retention will be measured using administrative indicators, including employment continuity during the study period, intention-to-leave responses, and self-reported likelihood of remaining employed at the hospital over the next 12 months.

### 2.12. Data Management

All completed questionnaires and surveys will be collected, securely stored, and coded to maintain confidentiality. Data will be entered into a database for analysis, with quality checks performed to ensure accuracy.

This systematic approach ensures that data collection is ethically sound, comprehensive, and consistent across all participants, providing a robust foundation for subsequent analysis.

### 2.13. Data Collection Instrument

This quantitative study will utilize a **self-administered structured questionnaire** designed to measure burnout, its contributing factors, and its relationship to nurse retention in the selected district hospital. The questionnaire is divided into four sections, each aligned with the study’s objectives and research questions.

**Section A** will collect **demographic information** such as age, gender, years of nursing experience, educational qualification, and employment category. These variables will enable the analysis of how personal and professional backgrounds relate to burnout and retention outcomes [21].

**Section B** will assess nurses’ **knowledge and awareness of burnout** and its effect on retention. Items will evaluate respondents’ understanding of burnout symptoms, risk factors, and the impact of burnout on personal well-being and workforce stability. This section’s questions are adapted from existing nurse retention surveys to ensure contextual relevance to the South African healthcare environment [22].

**Section C** will focus on **factors contributing to burnout** using the **Job Demands–Resources (JDR) model** as its theoretical framework. It will be operationalized through the **short version of the Burnout Assessment Tool (BAT)**, which measures exhaustion, mental distance, cognitive impairment, and emotional impairment [23]. The BAT’s focus on job demands (e.g., workload, staffing shortages, time pressure) and job resources (e.g., supervisor support, teamwork, autonomy) makes it highly suitable for capturing organizational factors that influence burnout and retention in the hospital setting.

**Section D** will assess **burnout’s impact on nurse retention and professional accomplishment** by integrating the **BAT short version** with selected items from the **Maslach Burnout Inventory (MBI) personal accomplishment subscale** [24]. This merged instrument approach addresses important measurement gaps: while the BAT provides a modern, comprehensive assessment of burnout dimensions consistent with the JDR model, it does not capture nurses’ positive feelings of professional efficacy and achievement. The MBI’s personal accomplishment subscale fills this gap by measuring nurses’ perceptions of their competence, career achievement, and impact on patient care, which are key predictors of retention.

By combining the BAT’s 12 core items covering exhaustion, mental distance, emotional impairment, and cognitive impairment with the MBI’s 3 personal accomplishment items, the questionnaire will capture both the negative and positive aspects of burnout. This integration also keeps the questionnaire concise, reducing respondent fatigue while maintaining strong content validity and allowing for historical comparability with prior nursing burnout research.

All responses will be recorded on a Likert scale to facilitate quantitative analysis. The instrument will undergo pilot testing with a small sample of nurses from a similar healthcare facility to ensure clarity, relevance, reliability, and validity before full-scale data collection. The Burnout Assessment Tool (BAT) short version contains 12 items across four dimensions, while the MBI personal accomplishment subscale includes 3 items. All items are rated on a 5-point Likert scale. Both instruments have demonstrated acceptable reliability and validity in nursing populations, including studies conducted in sub-Saharan Africa.

### 2.14. Data Entry and Analysis with Statistical Methods Used

Data analysis will be conducted using IBM SPSS version 29. Descriptive statistics (means, standard deviations, and frequency distributions) will summarize MBI scores and demographic characteristics. Inferential statistics will include chi-square tests to assess associations between categorical variables (e.g., burnout level and retention status), and independent t-tests or ANOVA for comparing mean burnout scores across different nurse categories or departments. Pearson correlation will be used to examine the relationship between MBI sub-scale scores and continuous patient outcome measures. Where appropriate, multiple regression analysis will be employed to identify predictors of nurse retention and burnout severity, controlling for confounding variables. Potential confounders such as age, gender, years of experience, professional category, and department will be controlled for in the multiple regression analysis.

Responses from the Burnout Knowledge and Response Survey will be analysed using descriptive statistics to summarize knowledge levels and awareness patterns. Frequencies and percentages will be used for categorical responses, while Likert-scale responses will be analysed using means and standard deviations. Cross-tabulations and chi-square tests will explore associations between burnout knowledge and factors such as department, years of experience, and retention intentions. Although Likert-scale data are ordinal, summed scale scores will be treated as approximately continuous variables, consistent with established practice in psychometric research, allowing the use of parametric tests where normality assumptions are met.

### 2.15. Data Management and Dissemination of Information

#### Data Management

All data collected during the study will be handled with strict confidentiality to protect participants’ privacy. Completed questionnaires and surveys will be collected in sealed envelopes or electronically stored in password-protected files to prevent unauthorized access. Each participant will be assigned a unique identification code to anonymize data and ensure confidentiality during data entry and analysis.

Data will be entered into a secure electronic database using software such as Microsoft Excel or SPSS version 30.0, with double data entry employed to minimize errors. Regular data quality checks will be conducted to identify and correct inconsistencies or missing information. Physical documents, such as consent forms and completed questionnaires, will be stored securely in a USB drive and will be backed up regularly on encrypted drives to prevent data loss. Data quality checks will include double data entry, range checks, and periodic audits to identify missing or inconsistent responses.

All data will be retained in accordance with ethical guidelines and institutional policies for a period of at least five years after project completion, after which they will be securely destroyed.

### 2.16. Dissemination of Information

The findings of this study will be disseminated through multiple channels to ensure broad access and impact. A comprehensive report will be prepared and shared with participating hospitals, health authorities, and stakeholders to inform policy and practice. Additionally, results will be submitted for publication in peer-reviewed journals focused on healthcare, nursing, and public health to contribute to the academic body of knowledge.

Presentations at national and international conferences will be organized to reach a wider audience, including policymakers, healthcare managers, and nursing professionals. Summary briefs and policy recommendations will also be developed to facilitate the translation of research findings into practical interventions. Furthermore, a community engagement session may be held to share key insights with the participating hospitals and the wider community, emphasizing the importance of addressing burnout and improving nurse retention in resource-limited settings.

### 2.17. Ethical Considerations

Permission of the Study: The proposal will be submitted to the Walter Sisulu University Ethics Research Committee for ethical clearance approval. Permission to conduct the study will be obtained from the Eastern Cape Department of Health and St. Elizabeth Hospital.

**INFORMED CONSENT:** Participants will be provided with comprehensive information about the study’s purpose, procedures, potential benefits and risks, and their voluntary participation. They will sign a written consent form indicating they understand the information and agree to participate freely. This process ensures informed and autonomous decision-making in alignment with current ethical research guidelines [8,24].

Confidentiality: All participant data will be anonymised, and no identifying details will appear in any dataset or report. Only the principal researcher and the academic supervisor will have access to sensitive information [24]. Data will be securely stored and encrypted to protect participants’ privacy and uphold their trust, in accordance with contemporary data protection standards [24].

Voluntary Participation: All participants will be explicitly informed of their right to refuse or withdraw from the study at any time without penalty or adverse consequences. This ensures full respect for participant autonomy, aligning with the core ethical principles governing human research [24].

Justice: Justice will be upheld through stratified random sampling to ensure fair representation of nurses from different departments, including emergency, maternity, general medical, and pediatrics. This method prevents bias or discrimination and ensures the findings reflect the diversity of nursing roles in the hospital [24].

Limitations: A key limitation of this study is its focus on a single district hospital, St. Elizabeth Hospital, which may restrict the generalisability of results to other hospitals or healthcare contexts in the Eastern Cape or South Africa. While this targeted approach allows for a detailed examination of nurse burnout in a high-pressure environment, it may not fully capture the diversity of experiences across multiple healthcare institutions. Due to the cross-sectional design, this study cannot establish causal relationships between burnout and nurse retention, but only associations at a single point in time.

## Data Availability

The data that support the findings of this study are available from the corresponding author upon reasonable request. Due to ethical and confidentiality restrictions, individual participant data are not publicly available, as they contain sensitive information about nurses employed at St. Elizabeth Hospital.

## References

[1] Kelly L., Gee P., Butler R. (2020). Impact of nurse burnout on organisational and position turnover. Nurs. Outlook.

[2] Khamisa N., Madala S., Fonka C.B. (2025). Burnout among South African nurses during the peak of the COVID-19 pandemic: A holistic investigation. BMC Nurs..

[3] Labrague L.J., De Los Santos J.A.A., Falguera C.C., Nwafor C.E., Galabay J.R., Rosales R.A., Firmo C.N. (2020). Predictors of nurses’ turnover intention at one- and five-year time points. Int. Nurs. Rev..

[4] Nursing Times (2022). Nurse Burnout: The Impact on Patient Care and Staff Retention. Nursing Times.

[5] South African Nursing Council (SANC) (2023). Provincial Distribution of Nursing Manpower Versus the Population of South Africa.

[6] National Department of Health (South Africa) (2023). 2030 Human Resources for Health Strategy.

[7] Munyewende P.O., Rispel L.C. (2014). Using diaries to explore the work experiences of primary health care nursing managers in two South African provinces. Glob. Health Action.

[8] Amin R., Anwar M., Anwar A., Kundi M.N. (2025). How severe is burnout among postgraduate medical trainees? A cross-sectional study at HMC, Peshawar. Prof. Med. J..

[9] Amin S.I., Mahdy R.S., El-Shafei D.A., Elmasry N., Eldawy H., MagdyAbdalla R., Fouad E. (2024). Burnout syndrome, anxiety, and depression symptoms among workers in radiation field. Middle East Curr. Psychiatr..

[10] Ashipala D.O., Nghole T.M. (2022). Factors contributing to burnout among nurses at a district hospital in Namibia: A qualitative perspective of nurses. J. Nurs. Manag..

[11] World Health Organization (2025). World Health Statistics 2025: Monitoring Health for the SDGs, Sustainable Development Goals.

[12] Griffiths K. (2022). Using restorative supervision to help nurses during the COVID-19 pandemic. Nurs. Times.

[13] McHugh M.D., Aiken L.H., Sloane D.M., Windsor C., Douglas C., Yates P. (2021). Effects of nurse-to-patient ratio legislation on nurse staffing and patient mortality, readmissions, and length of stay: A prospective study in a panel of hospitals. Lancet.

[14] Creswell J.W. (2018). Research Design: Qualitative, Quantitative and Mixed Methods Approaches.

[15] Maslach C., Jackson S.E. (1981). The measurement of experienced burnout. J. Organ. Behav..

[16] Brink H., Van der Walt C. (2006). Fundamentals of Research Methodology for Health Care Professionals.

[17] Aiken L.H., Cimiotti J.P., Sloane D.M., Smith H.L., Flynn L., Neff D.F. (2011). Effects of nurse staffing and nurse education on patient deaths in hospitals with different nurse work environments. Med. Care.

[18] Jun J., Ojemeni M.M., Kalamani R., Tong J., Crecelius M.L. (2021). Relationship between nurse burnout, patient, and organizational outcomes: Systematic review. Int. J. Nurs. Stud..

[19] Lake E.T., Sanders J., Duan R., Riman K.A., Schoenauer K.M., Chen Y. (2019). A meta-analysis of the associations between the nurse work environment in hospitals and 4 sets of outcomes. Med. Care.

[20] Rushton C.H., Batcheller J., Schroeder K., Donohue P. (2015). Burnout and resilience among nurses practicing in high-intensity settings. Am. J. Crit. Care.

[21] Schaufeli W.B., De Witte H., Desart S. (2020). Burnout Assessment Tool (BAT) Manual.

[22] Thomas J., Renter M. (2021). Improving Nurse Retention and Healthcare Outcomes: Innovating with the IMPACT Model.

[23] Van Bogaert P., Meulemans H., Clarke S., Vermeyen K., Van de Heyning P.H. (2009). Hospital nurse practice environment, burnout, job outcomes and quality of care: Test of a structural equation model. J. Adv. Nurs..

[24] Van Bogaert P., Timmermans O., Weeks S.M., van Heusden D., Wouters K., Franck E. (2014). Nursing unit teams matter: Impact of unit-level nurse practice environment, nurse work characteristics, and burnout on nurse-reported job outcomes, quality of care, and patient adverse events—A cross-sectional survey. Int. J. Nurs. Stud..

